# Decommissioning and Safe Disposal of Vaccine Cold Chain Equipment in Low- and Middle-Income Countries: Focusing on Processes, Risks, and Practical Challenges

**DOI:** 10.7759/cureus.100397

**Published:** 2025-12-30

**Authors:** Snehil K Singh, Dereje A Haile, Sabin Syed, Dheeraj Bhatt, Ghanashyam Sethy, Abdalla Hassan, Ajitendra Kumar, Sumeet Juneja, Bhrigu Kapuria

**Affiliations:** 1 Community Medicine, Private Practice, Nairobi, KEN; 2 Public Health, Private Practice, Nairobi, KEN; 3 Dentistry, Private Practice, Lilongwe, MWI; 4 Epidemiology and Public Health, Private Practice, Vientiane, LAO; 5 Epidemiology and Public Health, Private Practice, Lusaka, ZMB; 6 Immunization Supply Chain, Private Practice, Delhi, IND; 7 Epidemiology and Public Health, Private Practice, Sana’a, YEM

**Keywords:** beyond economic repair, cold chain equipment, condemnation, decommissioning, environmental health, health-care waste management, immunization supply chain, refrigeration equipment, safe disposal, vaccine cold chain

## Abstract

Immunization programs in low- and middle-income countries (LMICs) rely on large fleets of powered cold chain equipment (CCE), much of which is now aging, inefficient, or technologically obsolete. While substantial investments have expanded and modernized CCE inventories, formal frameworks for end-of-life management lag behind, and obsolete units frequently accumulate in facilities, yards, and warehouses. Inadequate decommissioning and disposal create environmental, occupational, programmatic, and financial risks that are poorly documented and weakly integrated into cold chain and healthcare waste management policies.

We conducted a narrative, process-focused review of electrical vaccine refrigerators and freezers in low- and middle-income settings. Normative guidance from global health, environment, and waste agencies was combined with national policies, program and evaluation reports, healthcare waste management assessments, and selected peer-reviewed literature. Sources were included if they described policies, procedures, or practices for decommissioning, removing, or disposing of vaccine CCE. Extracted information was organized along end-of-life lifecycle stages and synthesized narratively.

The review identifies a systems framework in which end-of-life CCE presents a complex hazard profile, including ozone-depleting and high-GWP (global warming potential) refrigerants, compressor oils, foams, electronics, and, for some solar units, batteries. Decommissioning can be structured into a governance setup followed by six operational steps from notification and technical condemnation through decontamination, removal, transport, and licensed final treatment, supported by cross-cutting documentation and reporting. When these steps are absent or weak, environmental and climate risks, occupational and community exposures, vaccine potency loss, *ghost* cold-chain capacity, and higher operating costs are common. Implementation is constrained by fragmented mandates, lack of dedicated financing and contract provisions, limited treatment infrastructure, weak inventories, operational and sociocultural barriers, and inequitable impacts on peripheral facilities and informal waste workers.

Decommissioning and safe disposal of vaccine CCE should be treated as routine lifecycle functions and explicitly embedded in cold chain improvement plans, contracts, financing arrangements, and information systems. Applying a structured, stepwise process within existing asset management, healthcare waste management (HCWM), and environmental frameworks can reduce safety and climate risks, improve use of space and resources, and close a long-neglected gap in immunization supply chain performance in LMICs.

## Introduction and background

Immunization programs in low- and middle-income countries (LMICs) depend on an extensive network of cold chain equipment (CCE) to keep vaccines potent. Because most vaccines are heat-sensitive, they must be transported and stored under strict temperature conditions, which demands robust and reliable CCE from the national store down to primary healthcare facilities [1]. In most LMICs, thousands of vaccine refrigerators, freezers, cold boxes, and carriers are deployed at clinics and health posts, forming the backbone of immunization delivery at the community level. This distributed CCE infrastructure is essential for reaching children with life-saving vaccines, but it also presents significant management challenges over the equipment lifecycle [2].

In the past decade, many LMICs have seen major waves of investment in vaccine CCE [3]. Partner-financed procurements and national replacement programs have introduced new generations of Performance, Quality, and Safety (PQS)-compliant refrigerators, freezers, solar direct-drive units, and remote temperature monitoring solutions into routine immunization systems. These deployments are intended to modernize the installed base and displace older, inefficient, or non-compliant equipment that has accumulated over several decades. At the same time, technical agencies and partners have issued standard operating procedures and guidance on CCE inventory management, preventive maintenance, and end-of-life management [4]. However, despite these advancements, many LMIC health systems continue to face gaps in routine maintenance and performance monitoring of equipment, delays in replacing ageing units, and inadequate planning for end-of-life management. While new procurements are helping to refresh cold chain capacity, the reality is that countries must also contend with decades’ worth of older CCE that is gradually deteriorating. In particular, the final stage of the lifecycle, decommissioning and safe disposal of obsolete equipment, has received little systematic attention [4].

These lifecycle challenges span all types of CCE, from large walk-in cold rooms to small vaccine carriers. A particularly problematic category is the legacy absorption refrigerator: older kerosene‑ or gas‑fueled vaccine fridges that were once ubiquitous and have since been largely phased out by newer technologies. Tens of thousands of these units were deployed in past decades; many are now nonfunctional and have been supplanted by solar or electric models [5]. Despite modernization and new procurements, many LMICs still lack routine end-of-life pathways for obsolete CCE. As a result, irreparable or outdated units are often retained in facilities, yards, and stores because decommissioning is not budgeted, roles are unclear, and compliant disposal pathways are not operationalized. This leads to equipment backlogs, weak inventory accuracy, and avoidable environmental, occupational, programmatic, and financial risks [2,4]. While this review focuses on electrical vaccine CCE (mains-powered and solar/battery-based units), absorption refrigerators are discussed selectively as legacy equipment still present in end-of-life backlogs, mainly to illustrate distinct hazard and disposal considerations that countries may face during decommissioning.

Failing to remove and safely dispose of obsolete CCE carries significant programmatic, safety, financial, and environmental risks. Programmatically, clutter from unused equipment can crowd limited storage space and complicate inventory management; worse, outdated units might be mistakenly used for vaccines, leading to temperature excursions and potency loss. There are also direct safety hazards [6]. Older absorption refrigerators contain ammonia and other pressurized gases that are toxic if released, and many aging compression-cycle refrigerators still contain legacy refrigerants such as CFCs and HCFCs that are controlled under the Montreal Protocol and its Kigali Amendment [7]. If these substances leak or are vented during informal scrapping or dumping, they pose health risks to technicians and communities and contribute to ozone depletion and climate forcing. Improper dismantling and disposal can also contaminate soil and groundwater through compressor oils, insulation foams, and electrical components. Financially, neglecting decommissioning wastes potential salvage value and resources, ties up staff time and storage space in managing defunct units, and may expose health authorities to penalties or remediation costs for noncompliance with environmental and waste regulations.

While World Health Organization-United Nations Children’s Fund (WHO-UNICEF) guidance (2018) provides important normative direction, this review adds value by synthesizing dispersed guidance, country documents, and program reports into a single, end-to-end operational framework for CCE end-of-life management. It extends beyond existing guidance by integrating systems framing (governance, roles, risk controls, documentation, and compliance) with implementation-oriented considerations and country experience signals to support practical adoption in low- and middle-income settings.

In this manuscript, end-of-life management refers to the full set of actions from decision-making through final disposition of CCE. Decommissioning refers to the planned, controlled process of withdrawing equipment from service and managing associated risks (e.g., electrical isolation and safe handling of refrigerants) before removal. Condemnation refers to the formal administrative decision that an asset is no longer fit for use and is authorized for disposal, transfer, or destruction. Retirement refers to the point at which equipment is permanently removed from the functional cold chain inventory. Disposal refers to the final disposition route (e.g., certified recycling, destruction, or environmentally sound disposal) consistent with national regulations.

Global policy and strategic context

Global immunization and health strategies increasingly recognize that supply chains must be not only reliable and efficient but also safe and environmentally responsible. The Immunization Agenda 2030 (IA2030) identifies supply chain and logistics as a core enabler of effective immunization and calls for investment in systems and infrastructure to safely manage, treat, and dispose of vaccine waste in order to reduce environmental footprints [8]. Gavi’s Phase 5 strategy (2021-2025) and the forthcoming Phase 6 strategy (2026-2030, “Gavi 6.0”) similarly emphasize strong, climate-resilient delivery systems as part of efforts to reach zero-dose and under-immunized populations and to support sustainable primary healthcare [9]. UNICEF’s Immunization Roadmap to 2030 further highlights the need to strengthen cold chain and stock management, improve distribution and waste management systems, and deploy climate-adaptive technologies in health facilities [10].

Beyond the immunization sector, global environmental agreements reinforce the importance of managing refrigerants contained in CCE. The Montreal Protocol has driven the phase out of ozone-depleting substances (ODS), and its Kigali Amendment establishes a timetable for phasing down hydrofluorocarbons, which are widely used as refrigerants in older refrigeration and air conditioning appliances, including health sector equipment [11]. National cooling plans and sustainable cooling guidance documents explicitly link implementation of the Kigali Amendment with promotion of low global warming potential (GWP) refrigerants and improved servicing and end-of-life practices for refrigeration equipment [12]. In this context, end-of-life management of vaccine CCE is directly connected to countries’ obligations on ODS and high GWP gases and broader agendas on green and climate-resilient health facilities.

Within this combined policy space, WHO and UNICEF issued dedicated guidance in 2018 on decommissioning and safe disposal of CCE, which sets out principles, roles, and stepwise procedures for planning and executing end of life management of CCE within national legal frameworks [4]. Healthcare waste management (HCWM) guidance for immunization programs and practical briefs on sustainable cold chains now reference this document as part of an integrated approach to waste, refrigerants, and environmental performance in immunization systems [13]. Taken together, these normative frameworks create an explicit expectation that immunization programs treat decommissioning of electrical CCE as a routine lifecycle activity that is embedded in national regulatory and planning processes, rather than as an ad hoc response once stores become congested or equipment fails catastrophically. However, implementation guidance remains fragmented across documents, and there is limited synthesis of practical experience on how countries are operationalizing decommissioning in routine practice.

Objectives of this review

This narrative review focuses on electrical vaccine CCE in LMICs in the post-Cold Chain Equipment Optimization Platform (CCEOP) period, with historical references included where relevant. Its objectives are to:

(1) Describe the main process elements that constitute decommissioning and safe disposal of electrical vaccine CCE, based primarily on WHO-UNICEF normative guidance and related technical materials.

(2) Identify and classify the principal risks that arise when decommissioning is absent or unsafe, with attention to environmental, occupational, programmatic, and financial dimensions.

(3) Synthesize the practical challenges that immunization programs face when attempting to implement decommissioning and disposal within existing regulatory, financial, and capacity constraints.

By organizing available evidence and practice insights around these dimensions, the review seeks to inform policy makers, logisticians, and technical partners who aim to embed end-of-life management of CCE within routine immunization supply chains, HCWM systems, and broader climate and sustainability agendas.

## Review

Methodology

Review Design and Purpose

We conducted a narrative, process-focused review to explore how vaccine CCE is decommissioned and disposed of in LMICs. The aim was descriptive rather than systematic: to map end-of-life processes, risks, and practical challenges for electrical vaccine refrigerators and freezers across the CCE lifecycle. Although the core scope is electrical vaccine refrigerators and freezers, we included limited discussion of absorption refrigerators as legacy equipment encountered in decommissioning backlogs, primarily to highlight hazard-specific considerations; the stepwise decommissioning framework presented is intended for electrical CCE.

A systematic review was not feasible for this topic because the evidence base is heterogeneous and predominantly consists of gray literature (guidance documents, policies, program reports, and country implementation materials) with limited comparable outcome measures. Therefore, we used a narrative approach to synthesize available sources into an implementation-oriented framework. The evidence base for CCE end-of-life management is largely normative and gray literature, with limited peer-reviewed empirical studies. Where available, we reference audits, assessments, evaluations, and country documentation to illustrate observed practice; otherwise, recommendations are presented explicitly as best practice.

In this review, condemnation is treated as the formal authorization step, retirement as removal from the functional inventory, decommissioning as the controlled withdrawal process, and disposal as the final treatment route.

Data Sources and Selection

We drew on a broad set of documents to capture both formal guidance and operational experience. Core sources were normative and technical guidance from global health and environment agencies, complemented by selected peer-reviewed publications, national policies and standards, program and evaluation reports, and healthcare waste management assessments. We also used immunization supply chain portals and document repositories to identify country and partner reports describing practical aspects of CCE removal, transport, dismantling, and disposal. Documents were identified through targeted searches of these repositories and by tracing references from key guidance and assessment reports.

We conducted targeted searches of PubMed/Google Scholar and key program portals (WHO, UNICEF, Gavi, TechNet-21, and partner repositories) using combinations of “cold chain equipment” and end-of-life terms (decommissioning, condemnation, disposal, retirement, waste management, refrigerant recovery, ODS). Approximately 50+ documents were screened, and 30 were included after relevance screening and full-text review for narrative synthesis. We used normative guidance and national policies as the primary framework and weighted country reports, audits/evaluations, and peer-reviewed studies to contextualize feasibility and implementation constraints, prioritizing recency and operational detail where evidence diverged.

Throughout the manuscript, we use *reported practice* for actions documented in country reports/audits/evaluations and *recommended best practice* for actions drawn from normative guidance, to clearly distinguish observed implementation from aspirational recommendations.

Inclusion Criteria and Scope

Sources were included if they provided substantive information on policies, procedures, or practices for decommissioning, removing, or disposing of vaccine CCE in LMIC health systems. We considered materials describing end-of-life decisions, inventory changes, physical removal from service, handling of refrigerants and other hazardous components, and final disposal or recycling. Electrical mains-powered, solar, and battery-based vaccine refrigerators and freezers were in scope. General e-waste or refrigeration literature without a clear link to health or immunization, and documents from high-income settings, were excluded. Recent materials were prioritized, but earlier guidance and assessments were used where they described legacy equipment types, such as absorption refrigerators, that dominate current decommissioning needs.

Analytical Approach

Given the process-oriented aim, we synthesized the information using a staged lifecycle lens. All extracted data (qualitative descriptions, recommendations, and any quantitative figures) were mapped to key lifecycle stages of CCE end-of-life: planning and decision-making for decommissioning, safe equipment deactivation and removal, environmental hazard mitigation (e.g., refrigerant recovery), and final disposal or recycling. Within each stage, we identify the actors involved (such as health facility staff, maintenance technicians, waste management contractors, and national program managers) and the specific process steps they undertake. We then compared and collated reported challenges and risks at each step, for example, safety hazards to workers, environmental risks like refrigerant leakage, regulatory or logistical hurdles, and resource or capacity gaps. This structured, thematic synthesis allowed us to highlight common practices and problem areas across sources. We did not quantitatively pool data; instead, we integrated findings narratively, triangulating between the guidance documents, case studies, and reports to develop a cohesive description of the decommissioning and disposal process in LMIC immunization programs.

Limitations

This review’s methodology has inherent constraints. First, it is a narrative synthesis without a systematic search or formal quality appraisal of sources. We relied on known repositories and expert-recommended documents rather than an exhaustive bibliographic search; thus, some relevant publications may have been missed. Second, the heavy reliance on gray literature (e.g., internal reports, guidelines) means some information is anecdotal or not peer-reviewed. While such sources offer practical insight, they may introduce bias and often lack the rigorous data of formal research. Third, there is a dearth of quantitative outcome data on CCE decommissioning in the literature - for example, few studies measure the environmental impact or cost-effectiveness of disposal interventions. As a result, our analysis is largely qualitative and process-oriented, synthesizing experiences and guidance rather than evaluating intervention outcomes. Finally, the scope was intentionally focused on LMIC settings and immunization supply chains; therefore, the conclusions may not generalize beyond those contexts. We acknowledge these limitations and present the findings as an informed narrative overview, meant to guide understanding and policy discussions rather than to serve as a systematic evidence review. The strengths of this approach lie in aggregating dispersed knowledge and highlighting practical challenges, but we advise caution in interpreting the findings in light of the above constraints.

Conceptual and systems framework

Lifecycle Stages and System Boundaries

The management of CCE spans a series of distinct lifecycle stages, from acquisition to retirement. Broadly, four major phases can be identified:

Planning and procurement: assessing needs, budgeting for purchase, and selecting appropriate equipment (via tender or donation)

Installation and commissioning: delivering the unit, preparing the site, installing and testing the device, and training staff in its use

Operation and maintenance: the long in-service period involves daily use for vaccine storage, routine temperature monitoring, preventive maintenance, and repairs to keep equipment functional.

End-of-life management (decommissioning and disposal): formally retiring the equipment, followed by removal, waste processing or recycling, and final disposal in compliance with environmental regulations [4]

For this review, the system boundary is drawn around the final stage - from the point at which a CCE is deemed obsolete or non-functional through decommissioning and final disposal. This focus is warranted because improper handling of end-of-life CCE can pose significant environmental and health hazards. Upstream stages (procurement, installation, and use) are considered only insofar as they inform or influence end-of-life management (for example, planning for decommissioning costs or training technicians in disposal practices during the equipment’s lifetime). The emphasis, however, remains on the processes and outcomes of the last phase of the CCE lifecycle.

Material and Hazard Profile at End of Life

Vaccine refrigerators and freezers contain a mix of materials that require careful handling at the end of life. Key components include refrigerant gases, compressor oils, insulating foam, and various electrical/electronic parts and metals. Refrigerants (historically CFCs and HCFCs, and now often hydrofluorocarbons (HFCs) or other alternatives), ammonia in absorption refrigerators, and polyurethane insulation foams are of particular concern - older formulations are ODS , and all refrigerants are potent greenhouse gases if released [8]. Similarly, the compressor oil and certain foam blowing agents can be toxic or flammable, posing local health and safety risks. The cabinet and cooling system incorporate metals (steel, aluminum, and copper) and plastics, along with wiring and circuit boards that may contain hazardous constituents such as lead, mercury, or flame retardants typical of electronic waste. If a solar CCE includes battery storage, spent batteries (lead-acid or lithium-based/cadmium) would add further hazardous waste to the profile, though many newer solar vaccine refrigerators use battery-free technology.

In waste management terms, an obsolete refrigerator or freezer from the cold chain is classified as waste electrical and electronic equipment (WEEE) and falls under hazardous waste categories. HCWM and e-waste regulations recognize these units as special waste streams requiring controlled disposal. For example, many countries mandate recovery of ODS refrigerants and other hazardous components before scrapping a refrigerator. National guidelines for medical equipment disposal often reference e-waste and hazardous waste laws, underscoring that end-of-life CCE must be handled within the frameworks for ODS chemicals, electronic waste, and other dangerous materials. In short, the mix of refrigerants, oils, foams, and electronic components in powered CCE represents a complex hazard profile at the end of life - one that demands specialized disposal processes to avoid environmental contamination and human exposure [14].

Actors and Regulatory Landscape at the Country Level

Managing the decommissioning and disposal of CCE requires coordination across multiple institutional players. In a typical LMIC context, the Expanded Program on Immunization (EPI) or logistics unit within the Ministry of Health is the primary owner of vaccine CCE and usually initiates the decommissioning process (e.g., deciding that a fridge is beyond repair). Biomedical engineers or refrigeration technicians are often responsible for assessing equipment condition, performing final shut-down or refrigerant recovery, and certifying that the unit is non-functional (*condemned*) [5]. Waste and environmental regulators, usually under a Ministry of Environment or an equivalent agency, establish the rules for hazardous waste and e-waste handling, such as requiring licensed disposal of refrigerants and compliance with environmental protection laws. These regulators may need to approve or oversee the disposal of CCE, although in practice health and environment sectors often operate in silos. Procurement and asset management teams (e.g., within the Ministry of Health or Finance) also play a role: they maintain asset inventories and may mandate how obsolete equipment is dispositioned (for instance, through public auctions, transfer, or destruction, in line with public asset disposal rules. Ideally, a formal authorized decommissioning committee brings these stakeholders together - including health logistics officials, engineers, procurement/finance officers, and environmental authorities - to review and approve each disposal action. Such a multi-sector committee can ensure that technical, financial, and regulatory requirements are all met before an old refrigerator is removed from service.

In practice, however, many LMICs face gaps and overlaps in this actor landscape. Responsibilities may be unclear or fragmented - for example, immunization programs might decommission units without informing environmental regulators, or vice versa. There are often gaps in policy and planning: a 2017 review of Gavi cold chain investments found that several countries had no finalized decommissioning and disposal plan or policy for replacing old CCE. As noted above, where funding, approvals, and licensed disposal pathways are weak, obsolete equipment is often retained on-site or in inventories beyond its useful life, and disposal may occur through ad hoc arrangements. Where disposal does occur, it may be ad hoc - sometimes handled by informal scrap traders or through one-time donor-driven cleanup campaigns - which can lead to unsafe practices like refrigerant venting or improper e-waste dumping [15]. This fragmented landscape indicates a need for stronger institutional coordination. Encouragingly, global guidance recommends integrating end-of-life management (including decommissioning and disposal) into CCE management plans; implementation remains context dependent and variably documented in publicly available reports. For instance, countries applying for new CCE support are urged to specify how old equipment will be safely decommissioned (including refrigerant removal and certified recycling), and some donors are considering earmarking funds to support proper disposal operations. Bridging the common silos between health logisticians, environmental regulators, and waste service providers is essential to ensure that the end-of-life phase of CCE is managed in a sustainable and legally compliant manner.

These lifecycle, hazard, and institutional dimensions together provide the systems lens through which subsequent sections examine decommissioning processes, risks, and implementation challenges.

Processes for decommissioning and safe disposal of CCE

End-of-life management of electrical vaccine CCE involves a sequence of planned activities that move obsolete units from initial identification to final treatment or reuse. These activities should protect program continuity, ensure sound environmental and safety practice, and comply with national asset-management and waste regulations [11]. In most LMIC settings, good practice can be organized into one governance step followed by six operational steps (Steps 1-6). Monitoring, documentation, and reporting run across all of them. Table 1 provides a detailed operational summary; for readability, key actions may be read as illustrative examples rather than an exhaustive checklist:

**Table 1:** Summary of decommissioning processes.

Step	Purpose	Key actions and actors (examples)
0. Governance and coordination	Establish a clear institutional framework before any field work starts, so that roles, decision-making authority, and budgets for decommissioning are agreed in advance. This prevents ad hoc decisions and ensures alignment with asset-management and environmental rules.	Ministry of Health convenes an authorized decommissioning or logistics sub-committee; EPI/cold-chain unit serves as secretariat; members from finance, environment, infrastructure, asset management, and partners; define lots and timelines, agree on tools and forms, link decommissioning with CCE replacement plans, and engage regulators early.
1. Notification and registration	Create an official record that specific CCE units are candidates for decommissioning, based on agreed criteria. This step brings obsolete equipment into a controlled pipeline and prevents silent accumulation in facilities.	Facility staff and technicians apply criteria (non-functional, beyond economic repair, obsolete, unsafe, surplus); complete a simple log with identifiers, location, condition, and reasons; attach photos where feasible; district and regional teams review, consolidate, and submit to the national level; record lost/stolen units through exception forms.
2. Technical assessment and condemnation	Verify the technical and financial case for retiring each unit and obtain formal authorization from the mandated asset body. This creates the legal and accounting basis for removal, transfer, or reuse.	Biomedical engineers or cold-chain technicians assess condition and options; compile a short report with equipment details, funding source, condition rating, and reason for removal; authorized committee (e.g., Board/Committee of Survey) reviews cases, may request further tests, and decides on condemn, transfer, reuse, or sale; issue condemnation forms and update cold-chain and asset registers.
3. Decontamination	Ensure the unit is clean and safe to handle before it is moved, reducing infection risk and meeting healthcare waste standards. This step separates basic hygiene from later technical dismantling.	Trained technician isolates power, defrosts if needed, removes all contents and loose parts; cleans and, where required, disinfects internal and external surfaces; dries and secures doors and shelves; labels the unit as decontaminated; technician and facility manager sign a short decontamination certificate.
4. Removal	Take the condemned unit safely out of service and transfer custody from the facility to the decommissioning process, without damaging the equipment or creating new hazards.	Management informs users that the unit is condemned and tags it “out of service”; the technician disconnects electrical and other connections, secures doors and components, applies hazard labels, and moves the unit to designated secure storage or the loading point; the cold-chain or asset manager updates the inventory and initiates or confirms formal write-off from the government asset register, with dates and references recorded in the transfer–disposal register.
5. Transport and transfer	Move condemned units from facilities to the authorized storage, treatment, or reuse site under documented chain-of-custody, minimizing the risk of leaks, accidents, or loss.	Cold-chain or asset managers arrange authorized transport; loaders keep units upright and strapped, with IDs visible; transporters carry transfer/disposal forms and manifests; receiving sites reconcile quantities and serials, sign delivery receipts, and complete intake logs; copies of documents are filed at facility, district, and national levels.
6. Final disposal and closure	Recover hazardous components, maximize safe reuse or recycling, and formally close the decommissioning lot in program and asset records. This step turns condemned units into properly managed waste and materials.	At licensed facilities, technicians reconcile loads, recover refrigerant into approved cylinders, drain compressor oil, mechanically disable units and dismantle into segregated streams (metals, plastics, electronics, batteries, PV components, foam); send streams to licensed recyclers or destruction facilities; manage solar and absorption units with specific procedures; issue certificates for recovery, recycling or destruction; update inventories and decommissioning databases; compile a lot-level close-out summary and report key indicators.

Cross-cutting systems: monitoring, documentation, and reporting

Across all steps, a simple but consistent documentation system is essential. Standard forms (notification, technical assessment/condemnation, decontamination certificate, transfer/transport manifest, and disposal certificate) should be used nationally so that every unit follows the same paper or electronic trail. Entries from these forms feed into a central decommissioning register linked to the CCE inventory and, where applicable, the government asset register. For each lot, the register records unit identifiers, locations, dates, decisions, storage periods, refrigerant recovery, and final treatment or reuse route [11]. Periodic summaries and an annual decommissioning report allow programmers and auditors to verify that condemned equipment has been removed from service, treated safely, and reported in line with environmental and public-finance regulations.

Risks associated with inadequate or unsafe decommissioning

Environmental and Climate Risks

Obsolete CCE contains ozone-depleting or high-GWP refrigerants, blowing agents in foam insulation, compressor oils, and electronic components. If units are dumped, vented, or burned instead of being properly decommissioned, these substances are released, contributing to ozone depletion, greenhouse‑gas emissions, and contamination of soil, water, and air [12]. HCWM guidance classifies such components as hazardous and calls for controlled recovery, treatment, and disposal [9].

Occupational and Public Health Risks

Haphazard dismantling exposes workers and nearby communities to acute and chronic hazards. Pressurized refrigerants can cause frostbite, asphyxiation in confined spaces, and, for flammable refrigerants, fire or explosion [13]. Informal e-waste handling without protective measures releases metals and persistent organic pollutants, increasing risks of respiratory disease, neurotoxicity, and cancer, particularly for children and pregnant women living near dumps or scrap yards [14]. Healthcare staff are also at risk if they handle end-of-life CCE without training or personal protective equipment, a concern highlighted in HCWM guidance.

Programmatic and Service Delivery Risks

Retaining aging, unreliable equipment in service increases the probability of temperature excursions (especially in kerosene-operated absorption refrigerators), freezing or heat exposure, and sudden breakdowns, with associated vaccine loss and missed immunization sessions. Assessments of cold-chain performance repeatedly show that poorly performing CCE is a major driver of potency loss and session cancellations [16]. Non-functional units left in facilities or stores consume scarce space and complicate layout and supervision, while *ghost* units still counted as functional in inventories distort cold-chain capacity estimates and misdirect planning.

Financial and Reputational Risks

Running old, energy-inefficient, or technologically obsolete CCE increases electricity or fuel costs and diverts limited maintenance budgets to uneconomic repairs. In many immunization programs, certain device families are regarded as obsolete regardless of age; for example, kerosene or gas-fired absorption refrigerators are generally excluded from vaccine use even when relatively new, yet may still occupy space and management effort [5]. Unsafe or non-compliant disposal can expose ministries of health to regulatory penalties and remediation costs, and damage confidence among donors and communities if harmful practices are publicised in the wider context of e-waste mismanagement. A weak record on responsible lifecycle management can also undermine credibility when programs seek further investment in cold-chain strengthening. Disaggregated cost ranges are rarely reported in the public domain and are often bundled within wider asset-disposal or e-waste contracts; costs therefore vary widely by logistics distance, equipment volume, and access to licensed recovery and treatment services.

Practical challenges in implementing decommissioning and safe disposal

Governance and Regulatory Fragmentation

Many countries lack a single procedure that links health-sector asset management with environmental and e-waste regulations. Immunization programs work under generic public asset and procurement rules, while environmental authorities oversee hazardous waste and refrigerants with limited coordination. Mandates for CCE decommissioning are spread across health, environment, and finance, which can slow or complicate condemnation and disposal decisions [17].

Financing and Procurement Constraints

End-of-life management is rarely budgeted explicitly. Program and grant funds focus on new equipment and maintenance, while collection, transport, decontamination, refrigerant recovery, and treatment are often unfunded. Procurement contracts frequently omit decommissioning obligations or take-back clauses, leaving disposal as a residual public-sector responsibility [4]. Where scrap revenue flows back to central treasuries, facilities, and districts have little direct financial incentive to invest effort in proper decommissioning.

Capacity and Infrastructure Gaps

Licensed facilities able to handle refrigerants, oils, and e-waste streams are often few and concentrated in major cities. Retrieving bulky CCE from remote health posts is logistically difficult and costly [18,19]. In many settings, cold-chain technicians are trained for installation and repair rather than hazard management, so safe dismantling, segregation, and documentation require additional training or contracted private providers [20]. Where formal recycling capacity is weak, informal actors fill the gap with limited safeguards.

Data and Information System Weaknesses

Inventory and asset-register systems often do not clearly distinguish functional, non-functional, and condemned units [21,22]. Facility stock frequently diverges from central databases, with obsolete units still listed as active assets. Maintenance, temperature-excursion, and asset-disposal records are seldom linked, and few systems track routine indicators on CCE decommissioning, such as numbers condemned, refrigerant recovered, or disposal route. This undermines planning, budgeting, and accountability.

Operational and Sociocultural Barriers

Health facilities and districts face competing priorities: organizing decommissioning lots, completing forms, and arranging transport are often seen as low-priority tasks [23]. Staff may fear audit or legal scrutiny when removing government assets, especially where procedures are unclear. Community or political resistance can arise if old refrigerators are removed before replacement units are visible. These factors encourage facilities to *park* obsolete CCE onsite for years rather than move it into formal decommissioning channels.

Equity and Ethical Concerns

Peripheral, hard-to-reach facilities are most likely to retain obsolete or unsafe equipment and least likely to benefit from organized collection campaigns. Where formal recycling capacity is weak, end-of-life CCE often enters informal scrap markets, shifting environmental and health risks to poorly protected workers and nearby communities. Children and pregnant women living around informal e-waste sites bear a disproportionate share of exposure to hazardous substances [24,25].

Combined Manifestations in Practice

In practice, countries experience these constraints in combination: unclear mandates, lack of budget, weak data, limited recycling options, and local resistance can all contribute to large backlogs of obsolete CCE, even where policies recognize the need for decommissioning [6]. Experience shows that programs make sustained progress only when decommissioning is treated as a routine lifecycle function, supported by explicit policy, financing, contracting provisions, infrastructure, and clear accountability, rather than as an occasional cleanup exercise.

Country experience and emerging practice on CCE decommissioning

Documented country experience indicates that the decommissioning of vaccine CCE is increasingly being approached as part of broader public-sector asset management, maintenance systems, and environmental compliance. In Zambia, the national process for disposal of obsolete and irreparable CCE is described as being routed through the Ministry of Works and Supply, initiated by a formal request and a Decommissioning Report, with equipment assessed and bonded for auctioning or destruction depending on status. The same source notes the role of the Zambia Environmental Management Authority (ZEMA) in regulating activities that may affect the environment, indicating alignment with national environmental oversight [26].

In Nigeria, national program documentation reflects efforts to institutionalize end-of-life management within routine cold chain systems. A 2021 country proposal reports that planned preventive maintenance guidelines were revised to include curative maintenance and procedures for decommissioning and disposal of obsolete CCE [27]. Nigeria’s national immunization strategy (NSIPSS 2018-2028) also explicitly references decommissioning of obsolete CCE in accordance with the Public Procurement Disposal Act, illustrating the linkage between cold chain lifecycle actions and public procurement and disposal rules [28].

In the United Republic of Tanzania, a program audit provides an example of how assurance mechanisms can surface practical priorities for system strengthening. The audit reports observations of non-functional and obsolete CCE stored at the Zanzibar Central Vaccine Store (Unguja) and recommends development and implementation of a decommissioning plan, alongside broader actions to strengthen CCE asset registration and management [29]. This illustrates how routine oversight processes can help translate end-of-life risk into actionable planning and accountability steps.

Multi-country evaluation evidence also points to emerging attention to decommissioning during platform-supported modernization. The CCEOP midline evaluation (Kenya, Pakistan, Guinea) reports that decommissioning planning was being drafted for older or malfunctioning equipment as part of efforts to improve system efficiency, and highlights decommissioning as a relevant element of overall systems strengthening linked to inventory visibility and lifecycle management [30].

## Conclusions

Decommissioning and safe disposal of powered vaccine cold chain equipment are core lifecycle functions, not optional clean-up activities, and are increasingly central to delivering IA2030, Gavi 5.0/6.0 priorities, and national climate and HCWM commitments. This narrative review highlights that, while countries have invested heavily in expanding and modernizing CCE fleets through initiatives such as the CCEOP, end-of-life management is not yet consistently institutionalized across the full asset lifecycle. As a result, end-of-life CCE is not consistently closed out through documented, safe, and compliant pathways.

The review proposes a structured, stepwise approach that links clear end-of-life criteria and governance with operational steps covering notification, technical condemnation, decontamination, removal, controlled transport, and licensed final treatment, supported by documentation and reporting; this is a synthesized framework derived from normative guidance and documented country experience, and it has not yet been formally evaluated for effectiveness. Importantly, country experience suggests there are workable entry points for operationalization, including public-asset disposal pathways, integration of decommissioning into preventive maintenance guidance, and the use of routine program assurance to prioritize and track corrective actions. These experiences reinforce that progress is feasible when roles, financing, contracting, and verification of final treatment are defined and enforced.

Priority actions for ministries of health and partners include embedding decommissioning within effective vaccine management (EVM) comprehensive immunization plan (cIP)/supplies chain improvement plan (SCIP) and HCWM plans, as well as within CCE contracts and grant conditions; securing dedicated financing and contracting models for end-of-life services; strengthening technician competencies and occupational safety requirements; investing in or securing access to licensed treatment chains; and improving inventory and information systems so that retired assets are rapidly removed, reconciled, and closed out with documented final disposition. Equity and ethical considerations, including the disproportionate burden of unsafe disposal on peripheral facilities and informal waste workers, should be explicitly addressed. If systematically implemented, decommissioning and safe disposal can improve vaccine storage reliability, reduce environmental footprints and financial waste, and close a long-neglected gap in immunization supply chain performance.
